# A case of hand urticaria, lip angioedema, and oropharyngeal pruritus induced by Japanese radish through IgE-mediated immediate allergic reaction

**DOI:** 10.1186/s13223-021-00538-1

**Published:** 2021-03-31

**Authors:** Sumiko Abe, Jun Ito, Sonoko Harada, Hitoshi Sasano, Shoko Ueda, Yuuki Sandhu, Tomohito Takeshige, Yoko Katsura, Norihiro Harada, Kazuhisa Takahashi

**Affiliations:** 1 Department of Respiratory Medicine, Juntendo University Faculty of Medicine and Graduate School of Medicine, 3-1-3 Hongo, Bunkyo-ku, Tokyo, 113-8431 Japan; 2 Clinical Research Center for Allergy and Rheumatology, National Hospital Organization, Sagamihara National Hospital, Kanagawa, Japan; 3 Atopy (Allergy) Research Center, Juntendo University Faculty of Medicine and Graduate School of Medicine, Tokyo, Japan; 4 Research Institute for Diseases of Old Ages, Juntendo University Faculty of Medicine and Graduate School of Medicine, Tokyo, Japan

**Keywords:** Japanese radish, Hand eczema, Oral allergy

## Abstract

**Background:**

Although Japanese radish (*Raphanus sativus *L.) is a common Japanese ingredient, there are few reports of IgE-mediated immediate food allergy caused by Japanese radish.

**Case presentation:**

A 48-year-old woman developed urticarial lesions on her hands after grating Japanese radish and also developed lip edema and oral itching when she ate a salad composed of raw Japanese radishes. Skin prick testing was positive to extract of grated Japanese radish. Moreover, immunoblotting analysis showed IgE reactivity in the patient’s serum to a single band at the 18 kDa in grated Japanese radish, suggesting that the heat-labile 18 kDa protein of raw Japanese radish may be a radish-specific antigen.

**Conclusions:**

To the best of our knowledge, this is the first case report of a patient with hand urticaria, lip angioedema, and oropharyngeal pruritus to raw Japanese radish through IgE-mediated immediate allergic reaction.

## Background

Radish (*Raphanus sativus* L.), belonging to the mustard family (Cruciferae or Brassicaceae), is a common edible vegetable; the taproot of white radish is widely consumed in East Asia, both fresh and dried. Although mustard allergy is a well-known food hypersensitivity in children [1], only few reports on IgE-mediated hypersensitivity reactions caused by radish are reported [2–6]. To our knowledge, this is the first reported case of hand urticaria, lip angioedema, and oropharyngeal pruritus caused by raw Japanese radishes.

## Case presentation

**Fig. 1:**
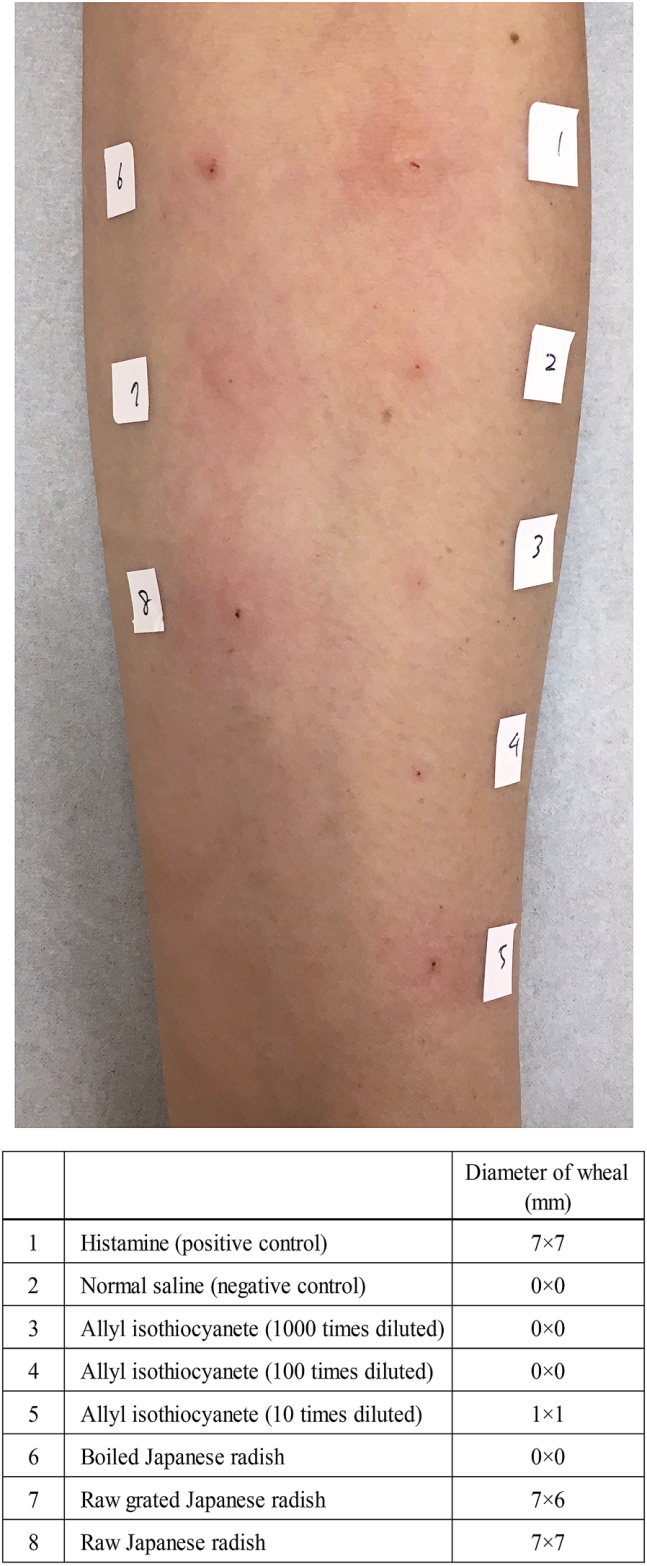
The skin prick test (SPT) was performed for extracts of the boiled and raw-grated Japanese radish (JR). The maximum wheal diameter was evaluated 15 min after the skin prick. Histamine and normal saline were used as positive and negative controls, respectively

A 48-year-old female desk worker presented to our outpatient department with a 3 year history of development of urticarial lesions on her hands while cooking a particular meal and lip swelling/oral pruritus during the same meal. She had no history of atopic diseases other than having unidentified hand eczema between the ages of 18 and 35. At the time of consultation, she realized that one of the causes of these symptoms was raw Japanese radish (JR). Within a few minutes of grating raw JR, she developed urticaria on her hands at sites of contact. Although no symptoms developed when she ate boiled JR, after eating a salad containing raw JR she reported immediate onset oropharyngeal pruritus. Lip angioedema developed within minutes of ingestion. These manifestations improved spontaneously after 1 to 2 h. The laboratory test showed that the serum total immunoglobulin E (IgE) level was 138 IU/mL. Serum specific IgE antibodies against house dust mites and cedar pollens were positive (chemiluminescence enzyme immunoassay class 2). Although the result of the skin prick test (SPT) for extracts of boiled JR and normal saline were negative (wheal diameter = 0 mm), a positive SPT was observed for the extract of raw JR and raw grated JR and histamine (wheal diameter ≥ 7 mm) (Fig. 1). Histamine and normal saline were used as positive and negative controls, respectively. To confirm that the reaction with the extract of raw and grated JR was not just an irritant reaction, we performed a similar prick test on healthy subjects and confirmed that the reaction was negative in healthy subjects. SPT results suggested that allergic reactions may be caused by extracts of raw-grated JR.

**Fig. 2:**
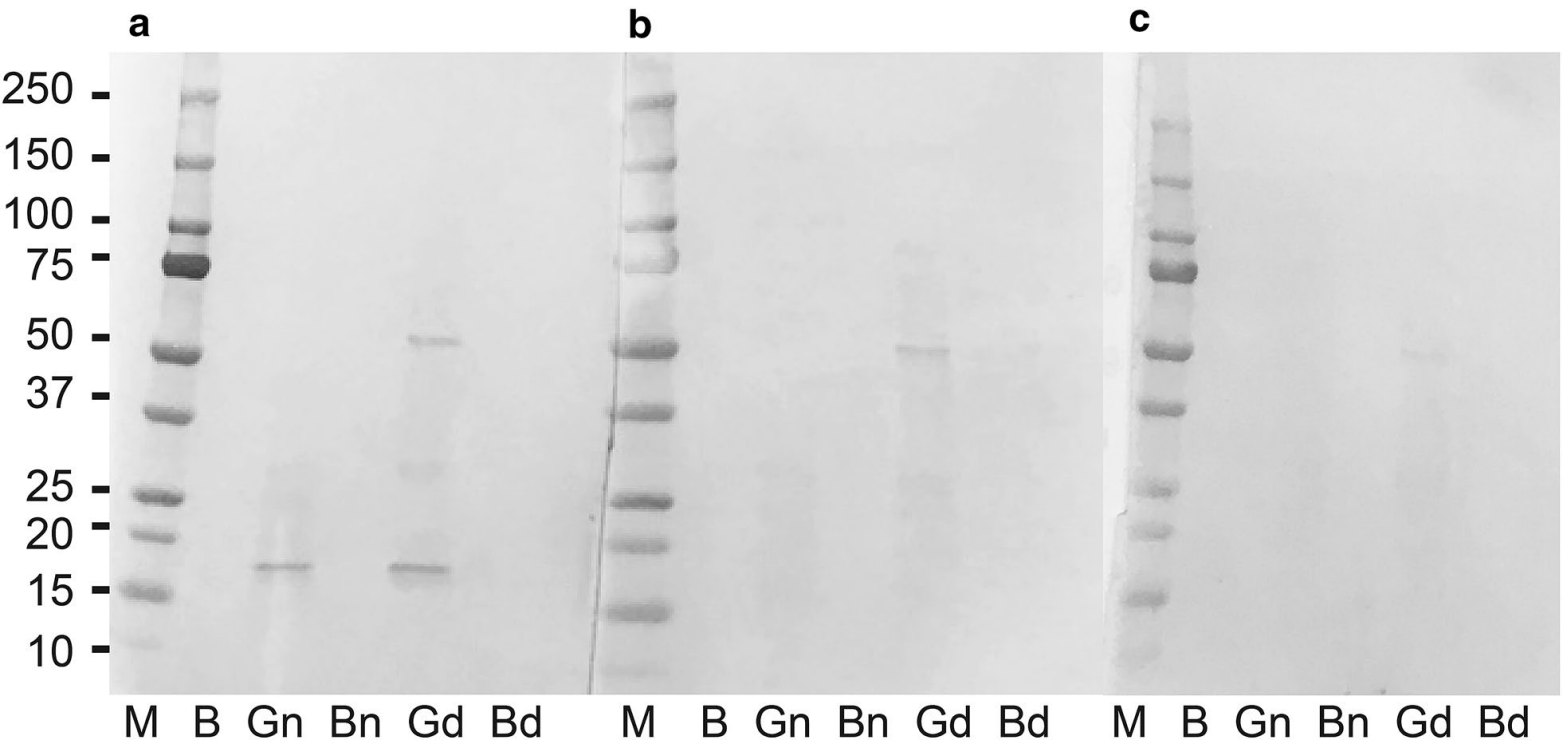
Immunoblot analysis of the raw-grated and boiled Japanese radish (JR). **a** The patient’s serum. **b** Control serum. **c** Inhibition immunoblots. Lane M, molecular weight marker; B, blank; Gn, grated JR under non-denaturing condition; Bn, boiled JR under non-denaturing condition; Gd, grated JR under denaturing condition; Bd, boiled JR under denaturing condition

The raw-grated and boiled JR taproots were ground by a musher until they became a paste and its supernatant was collected after centrifugation. Analytical electrophoresis was performed under non-denatured and denatured conditions of the proteins. Protein samples for denatured conditions were obtained by boiling with 2-mercaptoethanol and sodium dodecyl sulfate (SDS). Equal amounts of the extracts (50 μg) were separated by SDS–polyacrylamide gel electrophoresis and blotted onto polyvinylidene difluoride membranes. After blocking, blots were incubated overnight with the patient’s serum or control. The serum of patients with Japanese cedar pollen allergy was used as control. The membranes were then incubated with appropriate alkaline phosphatase-conjugated goat-anti human IgE antibody (used at 1/3000 dilution, Bethyl Laboratories, Montgomery, TX, USA), followed by detection with BCIP/NBT‐purple liquid substrate system for membranes (Sigma-Aldrich, St Louis, MO, USA). Immunoblotting analysis of non-denatured and denatured raw-grated radish showed IgE reactivity in the patient’s serum to a single band at 18 kDa and 2 bands at 18 and 65 kDa (Fig. 2a). However, immunoblotting revealed no band corresponding to IgE in boiled radish. In the control serum, immunoblotting analysis of denatured raw-grated radish revealed IgE reactivity corresponding to 65 kD (Fig. 2b). Inhibition immunoblots were also performed to detect IgE reactivity in the patient’s serum to the antigens in the proteins prepared from JR. In case of the experiment, non-denatured and denatured raw-grated radish taproot was each added to the patient’s sera 1 h before application to the blot membrane. The inhibition immunoblots revealed that the band at 18 kDa corresponding to IgE disappeared in case of raw-grated radish (Fig. 2c). These data suggested that the patent was sensitized to a heat-labile 18-kDa protein from raw-grated radish taproots.

## Discussion and conclusions

This is the first case of hand urticaria, lip angioedema, and oropharyngeal pruritus to raw JR through IgE-mediated immediate allergic reaction. Since 1974, 6 cases involving radish allergy have been reported in medical literature, besides the current case (Table 1) [2–7]. All the cases were of women with a median age of 46 (16–66) years. In 4 cases including the current case, dermatologic manifestations, oral allergy, and anaphylaxis were caused by radish (*Raphanus sativus* L.) while cooking and following a meal [2, 5, 6]. Two cases experienced urticaria and anaphylaxis after ingestion of food supplements containing black radish (*Raphanus sativus* L. var *niger*) [3, 4]. A Japanese article showed a case of allergic contact stomatitis caused for about 3 h after eating grated Japanese radish [7]. Five cases demonstrated positive SPT to the extract of raw radish, suggesting that the allergic reactions to radish may be due to IgE-mediated mechanisms [3–6]. However, the radish-specific antigen causing the allergy is unknown. Immunoblotting analysis in a previous case report demonstrated IgE reactivity in the patient’s serum corresponding to a 65-kDa protein in radish [5]. In the current case, immunoblotting analysis of denatured raw-grated radish revealed IgE reactivity corresponding to a 65-kDa protein in radish, in both patient and control sera; moreover, inhibition immunoblots revealed IgE reactivity in the patient sera to an 18-kDa protein in radish, but not to the 65-kDa protein. There are several important protein families of plant food allergens, including pathogenesis-related (PR)-10 and profilin. PR-10 and profilin have molecular weights of approximately 17–18 kDa and 12–15 kDa, respectively, and are not thermostable and are vulnerable to gastric digestion [8–13]. The PR-10 family includes Mal d 1 from apples and Bet v 1 from birch, and the IgE reactivity to PR-10 proteins is lost following heat treatment [11–13]. Although the 18-kDa protein in raw radish may be included in the PR-10 family due to molecular weight and its loss of antigenicity by heat, unfortunately, we have no information about this 18-kDa protein in radish. Moreover, radish belongs to the mustard family, it is well known that the mustard is identified as a food allergen and four allergens (*Sin* a 1, *Sin* a 2, *Sin* a 3, and *Sin* a 4) from yellow mustard (*Sinapis alba* L.) [14]. However, JR do not contain these allergens. Furthermore, in the current case, as a result of a detailed medical history interview focused on the possibility of cross-allergy to vegetables in the mustard family, raw wasabi (also denoted as Japanese horseradish), raw arugula, and raw cauliflower was also found to be responsible for her oral allergic symptoms. Therefore, further studies are needed to reveal whether the 18-kDa protein is a radish-specific antigen and these raw mustard family vegetables have an allergenic cross-reactivity with JR.

**Table 1:** Six cases of radish allergy

Case	Country	Age/sex	Food	*Raphanus sativus* L.	Symptom	SPT	Immuno blotting	Reference number
1	Canada	38F	Salad	*Raphanus sativus* L.	Contact dermatitis	ND	ND	[2]
2	France	16F	Food supplement	*Raphanus sativus* L. var. *niger*	Urticaria	+	ND	[3]
3	Portugal	56F	Food supplement	*Raphanus sativus* L. var. *niger*	Anaphylaxis	+	ND	[4]
4	Italy	66F	Salad	*Raphanus sativus* L.	Urticaria	+	+	[5]
5	Korea	46F	Kimuchi	Young radish (*Raphanus sativus* L.)	Anaphylaxis	+	ND	[6]
6	Japan	39F	Grated Jananese radish	Japanese radish (*Raphanus sativus* L.)	Contact stomatitis	ND	ND	[7]
7^*^	Japan	48F	Grated Jananese radish	Japanese radish (*Raphanus sativus* L.)	Hand urticaria, lip angioedema, and oropharyngeal pruritus	+	+	

*ND* no data, *SPT* skin prick test

^*^Current case

The mustard family vegetables, including JR, generate isothiocyanates, which are known as the pungent principle, through enzymatic hydrolysis of the corresponding glucosinolates [15]. Allyl isothiocyanate (AITC) is well known as the pungent principle of horseradish, wasabi, and mustard seeds, including black mustard (*Brassica nigra* L.), and is known to have antimicrobial activity against bacteria and fungi [16–18]. Although allergic contact dermatitis from AITC is well known, a previous retrospective study reported that 2 patients (0.8%) of 259 patients, who were suspected to have a contact allergy to food products, developed a positive allergic patch test [19]. The pungent principle of radish is not AITC, but 4-methylthio-3-butenyl isothiocyanate (MTBITC) [15], which is not the pungent principle of horseradish, wasabi, and mustard seeds. Unfortunately, because MTBITC is not commercially available, in the current case, the patient could not be extensively examined for other possible causes of her symptoms, including patch testing and SPT. Instead of MTBITC, SPT was performed using available AITC. Although only redness was observed at ten times dilution, the results of SPT for AITC were negative (Fig. 1). Therefore, it is unlikely that the symptoms of the current case were caused by an IgE-mediated allergic reaction to AITC.

Oral allergy syndrome (OAS) is defined as the symptoms of IgE-mediated immediate allergy localized in the oral and pharyngeal region and caused by contact with the acid and heat-labile antigen of raw fruits and vegetables [20–23]. Approximately 60% of food allergies are cross-reactivity between food and inhaled allergens, and the frequency of OAS in patients with pollen allergy was 5–8% [20, 21, 24]. Patients with hypersensitivity to pollen allergens have been found to have pollen food allergy syndrome (PFAS) which is clinically characterized by OAS symptoms immediately after food intake and is thought to occur when anti-pollen allergen IgE antibodies cross-react with the plant food allergens [21, 22, 25]. In the current case, our patient had specific IgE antibodies for Japanese cedar pollen, however, she had no symptoms of cedar pollinosis—including rhinoconjunctivitis. Moreover, it has been reported that patients with Japanese cedar pollinosis have PFAS caused by tomato fruit, but not radish [26, 27]. Mugwort-mustard allergy syndrome describes the association of mugwort pollinosis with several foods allergy from the mustard family [28–30]. However, this case did not have mugwort pollinosis and specific IgE antibodies against mugwort. Recently, the term PFAS has replaced traditional OAS, which may be not accurate because OAS not only responds to food allergens to cross-antigens but may also represent the clinical expression of primary sensitization to genuine food allergens [31, 32]. Therefore, her symptoms could be diagnosed as OAS, but could not be identified in the association with pollen in the current case.

In conclusion, to the best of our knowledge, this is the first report of a patient with hand urticaria, lip angioedema, and oropharyngeal pruritus to raw JR through IgE-mediated immediate allergic reaction. Immunoblotting analysis suggests that the 18-kDa protein of raw JR may be a radish-specific antigen.

## Data Availability

Data sharing is not applicable to this article as no datasets were generated or analyzed during this case report.

## Abbreviations

AITC: Allyl isothiocyanate
IgE: Immunoglobulin E
JR: Japanese radish
MTBITC: 4-Methylthio-3-butenyl isothiocyanate
OAS: Oral allergy syndrome
SDS: Sodium dodecyl sulfate
SPT: Skin-prick test
